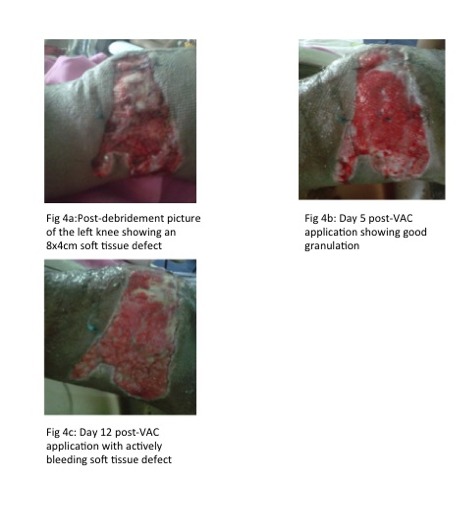# Improvised Suction Apparatus for Closure of Large Soft Tissue Deficit

**DOI:** 10.5704/MOJ.1307.002

**Published:** 2013-07

**Authors:** Kevin M Estillore, Gerto L Quevedo, Lauro R Bonifacio

**Affiliations:** Philippine Orthopedic Center, Maria Clara Corner Banawe Streets, Manila, Philippines; Philippine Orthopedic Center, Maria Clara Corner Banawe Streets, Manila, Philippines; Philippine Orthopedic Center, Maria Clara Corner Banawe Streets, Manila, Philippines

## Abstract

*Background:* Vacuum Assisted Closure (VAC) is an established method that can be used in order to cover for soft tissue defects. This study focuses on the use of a modified suction machine in the form of an aquarium pump as an economical substitute for the VAC dressing. The improvised suction apparatus was a regular aquarium pump, which was altered in order to draw air in instead of expelling it. The patients were followed-up for a period of three weeks in the wards. *Methods:* VAC was applied over the soft tissue defects. Sterilized foam was used and attached to a French 16 suction tube. A tight seal was ensured using a sterile adhesive covering. The tube was then attached to the modified vacuum machine, which maintained the negative pressure. *Case Studies :* Three cases are presented in this study. The first and second cases involved pediatric patients who sustained degloving injuries of the foot. The third case involved a 23 year-old male who sustained an open patellar fracture due to a motorcycle accident. The dressing was changed every five days and the progress was documented using photographs. The patients were placed on VAC dressing for a period of two weeks. On the 14th day, good granulation was evident on the soft tissue defects hence; the patients were scheduled and subsequently underwent splitthickness skin grafting. *Discussion :* The modified suction pump is an effective and economical alternative to the VAC machine for the purpose of providing constant negative pressure to promote tissue granulation and healing for large tissue defects. However, in order to establish the efficacy of the proposed intervention, the modified suction pump should be tested on more cases.

**Key Words:**

Vacuum-assisted Closure (VAC) System, modified VAC, aquarium pump, soft tissue defect coverage, alternative negative pressure source

## Introduction

Vacuum-assisted closure (VAC) was first investigated by Michael Morykwas, and Louis Argenta in 1997 using wounds created on swine subjects.

It is a development from the standard surgical procedure, which uses vacuum-assisted drainage to remove blood or serous fluid from an operation site in order to provide a drier surgical field and to control blood flow. Possible reasons for wounds to heal quicker with VAC therapy partly because removal of exudates and reduction of periwound edema, increased tissue perfusion, formation of granulation tissue, reduction of complexity/ size of wound, optimization of wound bed prior to or following surgery, and creation of a closed, moist environment thereby supplying the wound with oxygen and nutrition to promote accelerated healing 1.

The classic VAC system was composed of a vacuum pump (negative pressure unit), canister, tubing which connected the dressing to the pump, and a VAC dressing pack (foam and occlusive drapes). Various modifications have been made on the original system depending on the availability of materials and applicability to certain cases.

Negative pressure environment is only feasible with the availability of a suitable negative pressure source. Dressing materials, tubings, and reservoir bottles can easily be procured. The most technically-challenging part of the process would be getting the right kind of pump which would maintain the negative pressure for long hours of use. An aquarium pump is recommended because it can easily be found. It is cheap £5 (US$6.50) and is designed for continuous use for weeks or months on end using minimal electrical power and emitting minimal noise.

## MODIFIED SUCTION APPARATUS (AQUARIUM PUMP)

**Fig. 1a - 1h:**
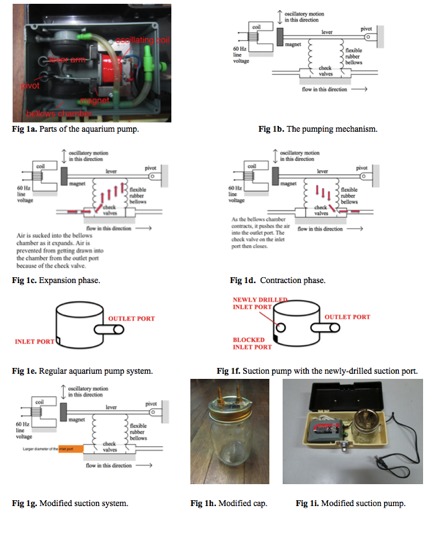


The idea of a modified suction machine using an aquarium pump was proposed by Kim Jingco 2. He suggested modifying the mechanism of an aquarium pump to make it suck air in rather than blow it out. The basic parts of a regular aquarium pump are discussed as follows (Fig 1a).

1. Oscillating coil – this serves as the pump’s main motor. It generates an electromagnetic field that switches polarity fifty to sixty times a second. The magnetic field causes the lever to swing to and fro, attracting and repelling the magnet at the end of the lever arm.

2. Pump lever – this has a pivot/ counterbalance on one end and a magnet on the other. The magnet is repelled and attracted by the oscillating magnetic field, which causes the lever to swing rapidly from side to side. This makes the bellows chamber expand and contract alternately.

3. Bellows chamber – this is a compartment that receives air from the inlet tube and releases air into the outlet tube.

4. Check valves – these serve as one-way panels that prevent air from going in the opposite direction.

The bellows chamber expands during the expansion phase (Fig. 1c) and in the process, sucks air into the pump via the suction or inlet hole. One-way valves are present to prevent air from getting drawn in via the outlet port. As the bellows chamber springs back in during the contraction phase (Fig. 1d), the compartment becomes smaller, which causes the air to be pushed into the outlet port.

The typical aquarium pump (Fig. 1e) has a cylindrical tube that serves as its outlet port, and a slit-like opening at the opposite end that serves as its suction port. In order to convert it into a suction machine, a new suction inlet should be placed and sealed properly (Fig. 1f). The old suction slit should be covered in order to prevent other ports of air entry. The new suction port should be placed in an area where it will not get in the way of the other parts of the machine. Using a permanent pen, mark the spot where the new suction port will be placed. Remove the whole pump and drill an appropriately-sized hole on the pump body. Use a small drill bit to increase the size of the hole progressively then use larger drill bits until the right size is obtained. Use an adhesive glue to cover the original suction port.

For exudates and secretions, use a regular glass jar to serve as a container (Fig. 1h). Place outlets on the cap of the jar to serve as attachments for suction tubings.

## Case 1

**Fig. 2a - 2h:**
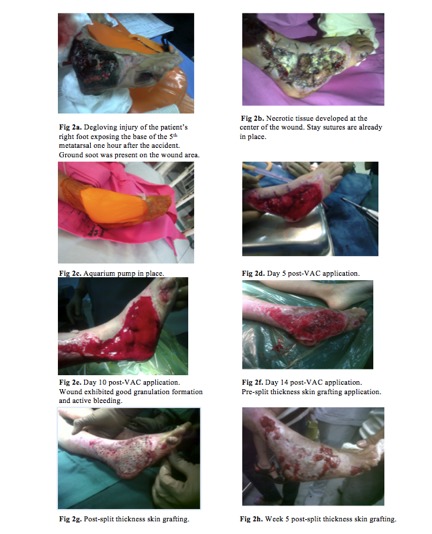


The patient was a seven year-old male who sustained a degloving injury on his right foot caused by a motor vehicle accident (Fig. 2a). No bony structures were involved, although the base of the fifth metatarsal was exposed. Immediate flushing and debridement was done at the emergency room.

Flushing was done daily using third’s solution (10cc bleach, 10cc cane vinegar, 80mL Plain Normal Saline Solution or PNSS). After one week of using the same solution to clean the wound, there was minimal granulation developing on the surface of the defect. Necrotic tissue (Fig. 2b) was also developing at the center of the wound therefore a repeat debridement was scheduled.

To promote better granulation, VAC application was considered. In this case, the author made use of an aquarium pump designed to maintain negative pressure over the wound (Fig. 2c). The aquarium pump was an apparatus being studied by our trauma team and this particular case was documented as our pilot case.

VAC was applied on the anterolateral aspect of the patient’s right foot. Sterilized foam was used and attached to a French 16 suction tube. A tight seal was ensured using a sterile adhesive covering. The tube was attached to the modified vacuum machine which maintained the negative pressure. The parent was instructed to strictly adhere to the schedule of one hour on, one hour off. The dressing was changed every five days and the progress was documented using photographs.

Fourteen days after using the modified aquarium pump, there was evidence of good granulation and active bleeding. The patient then underwent split-thickness skin grafting (Fig. 2g), and discharged from hospital. The patient’s parent was instructed to follow-up at the out-patient department after one week for wound inspection and care.

## Case 2

**Fig. 3a - 3d:**
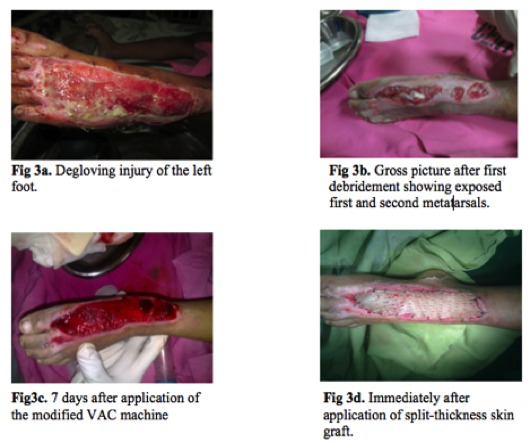


The patient was a 10 year-old male who sustained a degloving injury of the left foot because of a motor vehicular accident (Fig. 3a). The first and second metatarsals were exposed on the dorsum of the foot after the first debridement (Fig. 3b). The patient was indicated for application of modified VAC to promote better granulation.

On the first dressing change seven days after the application of VAC, there was good granulation tissue over the soft tissue defect (Fig. 3c). VAC therapy was continued for another seven days and the patient was subsequently scheduled for split-thickness skin grafting (Fig. 3d).

## Case 3

The patient was a 23-year-old male who sustained an open left patellar fracture after crashing into the pavement while riding his motorcycle. After the initial debridement, there was an 8x4cm soft tissue defect on the lateral aspect of the left knee (Fig. 4a). The modified VAC system was applied to reduce the complexity and size of the wound.

During the change of dressings five days post-application of the negative pressure dressing, there was good granulation over the soft tissue defect (Fig. 4b). The second change of dressings on the 12th day showed actively bleeding soft tissue on the left knee (Fig. 4c). He was then scheduled for split-thickness skin grafting later.

## Discussion

In VAC therapy, the application of topical negative pressure (vacuum) removes blood and serous fluid, reduces infection rates (closed/ sealed system creates a hypoxic environment) and improves localized blood flow, which in turn increases oxygen supply to the affected area for better wound healing. Leininger et al. conducted a study involving the use of VAC therapy in high-energy wounds. In their case series involving 88 patients, wound VAC resulted in earlier, more reliable primary closure of soft tissue defects 4.

A systematic review done by Pham et al. 5 enumerated the sequence of procedure for VAC application. Firstly, wound irrigation, adequate debridement and hemostasis should be done. An open-pore, reticulated medical-grade foam was recommended as it is most effective at transmitting mechanical forces across the wound and provides an even distribution of negative pressure over the entire wound bed to aid in wound healing. In our local setting, however, sterilized foams are normally used because of economical reasons and availability. Embedded in the foam is a suction tube that is connected to a vacuum pump that has a collection canister.

The foam is sealed using an adhesive drape (e.g. Opsite, Ioban). The drapes should cover at least three to five centimeters (3-5 cm) of healthy tissue to ensure a seal. According to the Principles of Best Practice: Vacuum- Assisted Closure dressings should be changed every 48-72 hours, dependent upon the patient’s condition. However, they recommended that the change of dressing should not be less than three times a week . Application of the negative pressure dressing using the aquarium pump follows the same sequence.

The use of a modified VAC machine in the form of an aquarium pump is highlighted in this study. The aquarium pump provides a low-cost negative pressure source, compared to suction pumps that are normally used in the wards. Other parts of the machine (i.e. foam, tubings, bottles) can be easily procured and safely sterilized. According to the Policy for the Management of Vacuum Assisted Closure (VAC) Therapy published by the Royal United Hospital Bath, all VAC systems are charged at a rate of £39 (US$50) per day. If the patient is at a hospital and requires VAC therapy during his or her stay, this would prove to be rather costly.

Other concerns regarding the efficacy of VAC therapy include the amount of vacuum pressure and duration of vacuum therapy. Blood flow studies using Doppler ultrasound found that blood flow to a wound peaked at 125mmHG and gradually decreased at settings greater than 125mmHg, with blood flow falling below the baseline measurements observed at room pressure at 400mmHg. The blood flow increased again with the re-establishment of a vacuum with an optimum cycle of 5 minutes on and 2 minutes off 1. Other sources, on the other hand, have different recommendations regarding the clinical uses of VAC therapy. Reduced pressures of 50-75 mmHg are used for larger cavities or acute traumatic injuries.

Instead of utilizing the normal outward flow of air seen with regular aquarium pumps, we had reversed the course of airflow of our apparatus in order to have a suctioning effect. The pump pressure was measured using an aneroid gauge from a sphygmomanometer which was attached to the aquarium pump. According to a pilot measurement of the amount of vacuum pressure provided by the aquarium pump, it was recorded to be at 120-125mmHg.

Edema can compress vascular and lymphatic drainage from a wound. With the use of VAC, excessive fluid is removed therefore restoring vascular and lymphatic flow. Wounds treated with VAC have demonstrated to require fewer courses of antibiotics compared to conventionally-treated wounds. Reduced levels of bacteria have also been demonstrated experimentally and clinically in VAC-treated wounds 1.

For wounds with large soft tissue defects, daily wound care is necessary, involving daily wound flushing and dressing. For these types of injuries, the use of vacuum-assisted closure is more cost-effective because less dressing changes are required. Since the VAC dressing is changed only two times or three times a week, wound care costs are substantially lower. Also, less frequent dressing changes result in increased patient comfort. This would prove to be more advantageous especially for the pediatric population wherein dressing changes could lead to added anxiety and distress to patients.

In our opinion, the modified suction/ aquarium pump is an effective and economical alternative to the VAC machine in providing constant negative pressure to promote tissue granulation and healing for large tissue defects. Parts of the modified pump may be easily and cheaply-procured, which will be beneficial to patients seen at our local setting.

**Fig. 4a - 4c:**